# Impact of Spin-Orbit Torque on Spin-Transfer Torque Switching in Magnetic Tunnel Junctions

**DOI:** 10.1038/s41598-020-59533-y

**Published:** 2020-02-18

**Authors:** Sachin Pathak, Chanyoung Youm, Jongill Hong

**Affiliations:** 1 Materials Science and Engineering, Yonsei University, Seoul, 03722 Korea; 2 Present Address: Physics, University of Petroleum and Energy Studies, Dehradun, 248007 India

**Keywords:** Magnetic devices, Magnetic properties and materials

## Abstract

The paper presents our simulated results showing the substantial improvement of both switching speed and energy consumption in a perpendicular magnetic tunnel junction (p-MTJ), a core unit of Spin-Transfer-Torque Magnetic Random Access Memory (STT-MRAM), by the help of additional Spin-Orbit-Torque (SOT) write pulse current (WP_SOT_). An STT-SOT hybrid torque module for OOMMF simulation is implemented to investigate the switching behavior of a 20 nm cell in the p-MTJ. We found that the assistance of WP_SOT_ to STT write pulse current (WP_STT_) have a huge influence on the switching behavior of the free layer in the p-MTJ. For example, we could dramatically reduce the switching time (*t*_SW_) by 80% and thereby reduce the write energy over 70% as compared to those in the absence of the WP_SOT_. Even a very tiny amplitude of WP_SOT_ (*J*^SOT^ of the order of 10^2^ A/m^2^) substantially assists to reduce the critical current density for switching of the free layer and thereby decreases the energy consumption as well. It is worth to be pointed out that the energy can be saved further by tuning the WP_SOT_ parameters, i.e., amplitude and duration along at the threshold WP_STT_. Our findings show that the proposed STT-SOT hybrid switching scheme has a great impact on the MRAM technology seeking the high speed and low energy consumption.

## Introduction

Magnetic Random Access Memory (MRAM) has known to be an outstanding candidate among next-generation memories due to its various advantages, such as non-volatility, high-speed operation, high density and scalability, over other competing memories^1–4^. In particular, spin-transfer torque MRAM (STT-MRAM) composed of perpendicular magnetic tunnel junctions (p-MTJs) has received a significant attention because it offers reduced write current and strong thermal stability^5^. In an MTJ, there are two ferromagnetic (FM) layers separated by an insulating tunneling barrier. One FM layer has a fixed magnetization and another has a variable magnetization (called as a free layer) which can be made to align either parallel (P) or anti-parallel (AP) with respect to the fixed layer. Magnetization of the free layer is used to store the data and can be switched by spin-polarized electrons (equivalently spin current) without a magnetic field. When the spin-polarized current flows through the free layer, the layer absorbs spin angular momentum from the electrons and as a result, its magnetization flips, which is the reason why we call it spin (momentum) transfer torque. STT-MRAM faces various challenges along with its merits such as, the reliability of a tunnel barrier, long write latency and small energy efficiency due to still high write current. Out of these, the most important issue which needs to be counter first is high energy consumption due to high write current and long write latency. The current density for switching of STT-MRAM is relatively large and hence large transistors are inevitable to drive it, which thus significantly limits their future use for memory applications^6,7^. The sustainability of higher switching current density of the tunnel barrier also raises reliability issues and leads to the degradation of related MTJ performance, such as, tunnel magneto resistance (TMR), write current margin, and write speed on the time span^8–10^. The situation will be even much worse when further scaling of STT-MRAM enters into a nanometer regime.

Various schemes are introduced to overcome these obstacles. For example, they are the application of a manipulated write pulse current and the use of voltage control magnetic anisotropy (VCMA) or spin-orbit torque (SOT) with the assisting STT^11–14^. All of these schemes are gaining great attention equally in recent times. In our previous study, we found a way to save the energy by using an overshoot transient pulse in the case of STT switching^12^. The energy could be saved up to 9%. However, it is still high for applications. The electric field (E-field) switching scheme is promising to significantly reduce the energy since the energy barrier for magnetic switching can be reduced through the VCMA effect. A significant reduction of switching current by two orders of magnitude was reported by combining the E-field effect to STT^11,15^. In spite of those advantages, VCMA-STT requires delicate pulse engineering as it requires two-step pulses. On the other hand, SOT switching is also gaining interest in order to overcome the above mentioned problems with STT-MRAM^16,17^. SOT composed of two orthogonal torques originated from the Rashba effect and the Spin-Hall effect (SHE) uses an in-plane current to reverse the state of the free layer without passing a current through the tunnel junction and separates the writing path from the reading path. Separate read and write lines in SOT-MRAM promises strong reliability^18,19^. What makes it better is that the torque generated by SHE achieves direct switching since there is no counter-acting torque unlike STT. Therefore, SOT can switch the magnetization faster than STT, which makes MRAM operation speedy and energy-effective. In spite of such excellent attributes, SOT switching itself provides stochastic, which needs to be a breakthrough for deterministic. Since, SOT-MRAM provides the reliable, energy efficient and fast memory technology solution; it has emerged as a strong contender, but its stochastic nature comes out as a big disadvantage which makes it difficult to utilize in practical devices though a couple of solutions have been suggested to make the switching deterministic^13,20,21^. In addition, increasing an spin-Hall angle or reducing SOT switching current is still a challenge for the application of the SOT switching scheme into MRAM. Conclusively, none of the above phenomena (nor SOT neither STT) are ready to overtake solely in order to employ for the realization of the storage devices at this current stage of research and development. However, one has to complement to another for better performance of p-MTJ. One can use SOT to assist STT switching in MRAM for the improvement of write speed and energy saving which is the strong motivation of this research. Numerous studies have reported the combining effect of SOT and STT switching for applications^13,14,21–25^. Some of them have focused to make SOT switching to be deterministic by applying the STT current^21^ or the alternating on/off pulse current of SOT and STT^13^. Here, we propose a new write scheme for an MTJ mainly by STT pulse current with the help of SOT pulse current in order not only to reduce the energy but to gain the switching speed by means of micro-magnetic simulations, where tiny SOT current have a great impact on the STT switching characteristics.

In this article, we are combining both of the aforementioned phenomena in a 3-terminal MTJ device. We introduce a new OOMMF extension module based on the STT switching with the assistance of SOT in p-MTJ cells. Our modified module consists of an SOT term in addition to the Landau-Lifshitz-Gilbert (LLG) ordinary differential equation with an STT term. This module is developed to investigate the magnetization dynamics of a free layer in the influence of STT write pulse current (WP_STT_) and SOT write pulse current (WP_SOT_), simultaneously. In this study, we compared the MTJ switching by WP_STT_ and WP_SOT_ for the p-MTJ with a cell size of 20 nm. Using our hybrid write scheme, the energy consumption can be dramatically reduced with the assistance of tiny WP_SOT_ to WP_STT_ for switching of magnetization of the free layer.

### STT-SOT hybrid torque model

An STT-SOT hybrid OOMMF module uses a time evolver that integrates the LLG equation with the STT and an additional SOT term, which governs the current induced magnetization dynamics of a free layer^26–29^. Spin-orbit torque (${\overrightarrow{\tau }}_{{\rm{SOT}}}$) is incorporated as a new torque term along with spin-transfer torque (${\overrightarrow{\tau }}_{{\rm{STT}}}$) in the ordinary differential equation to optimize the effect of additional torque on the magnetization for switching due to SOT (presented in Eq. 1).

1 $$\frac{d{\overrightarrow{m}}_{free}}{dt}=-\,\gamma {\overrightarrow{m}}_{free}\times {\overrightarrow{H}}_{eff}+\alpha {\overrightarrow{m}}_{free}\times \frac{d{\overrightarrow{m}}_{free}}{dt}+{\overrightarrow{\tau }}_{STT}+{\overrightarrow{\tau }}_{SOT}$$

2 $${\overrightarrow{\tau }}_{STT}=-\,\gamma {a}_{J}{\overrightarrow{m}}_{free}\times ({\overrightarrow{m}}_{free}\times {\overrightarrow{m}}_{fixed})-\gamma {b}_{J}({\overrightarrow{m}}_{free}\times {\overrightarrow{m}}_{fixed})$$

3 $${\overrightarrow{\tau }}_{SOT}=-\,\gamma {\tau }_{S}{\overrightarrow{m}}_{free}\times ({\overrightarrow{m}}_{free}\times \overrightarrow{\sigma })-\gamma {\tau }_{F}({\overrightarrow{m}}_{free}\times \overrightarrow{\sigma })$$

4 $${a}_{J}=\eta \frac{\hslash {J}^{STT}}{2e{\mu }_{0}{M}_{S}{t}_{F}}\,{\rm{and}}\,{\tau }_{S}={\theta }_{SO}\frac{\hslash {J}^{SOT}}{2e{\mu }_{0}{M}_{S}{t}_{F}}$$

where, *η and θ*_SO_ are the spin torque efficiency and spin orbit torque efficiency, respectively.

**Figure 1:**
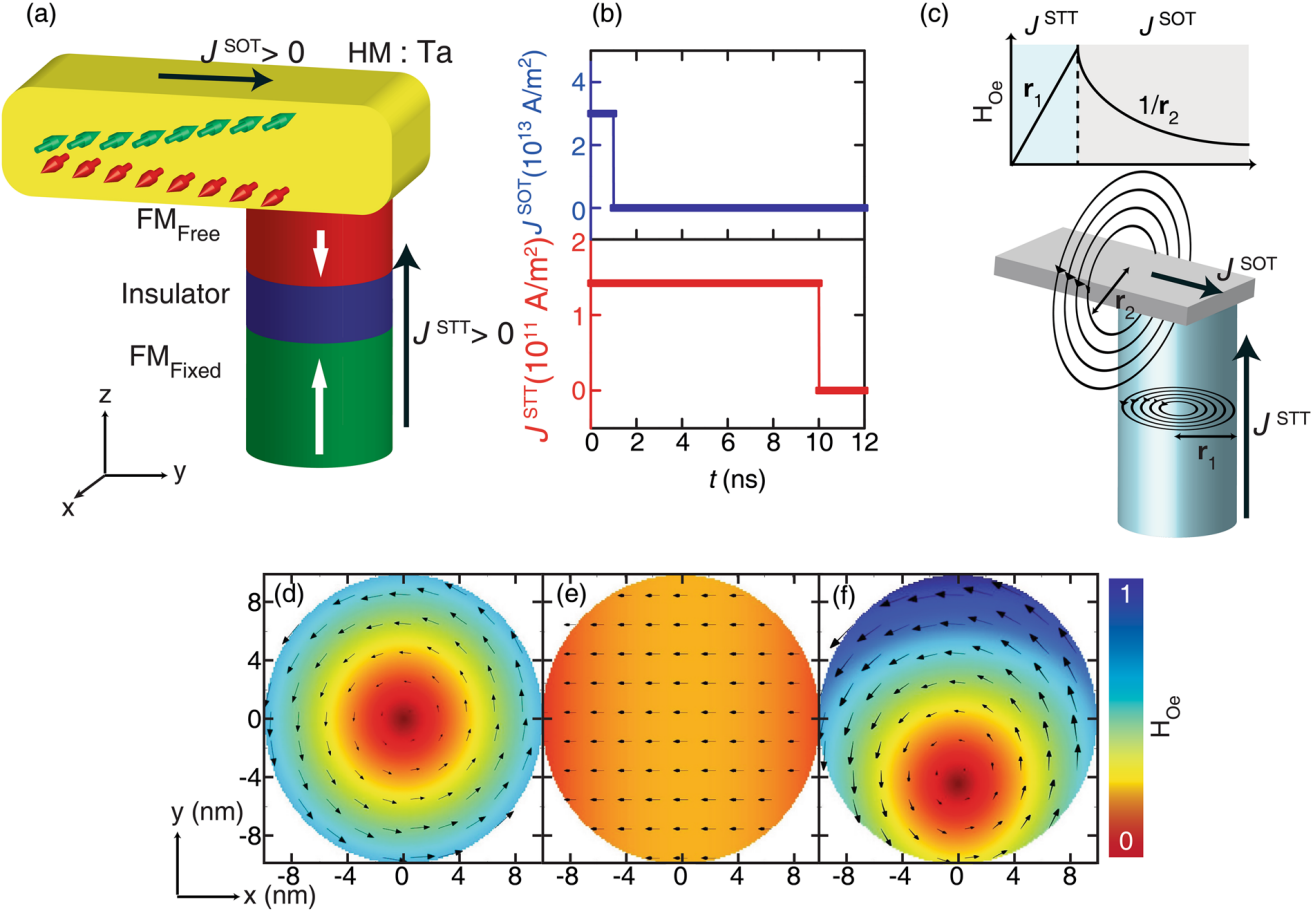
(**a**) A schematic of a hybrid p-MTJ cell composed of non-magnetic polarizing layer/FM (Ferromagnet)/I (Insulator)/FM with a cell diameter of 20 nm. (**b**) Design of hybrid write pulse of *J*_c_^STT^ = 1.42 × 10^11^A/m^2^ for 10 ns and *J*_c_^SOT^ = 3.0 × 10^13^A/m^2^ for 1 ns. (**c**) The spin current is accompanied by a charge current which gives rise to Oersted fields inside and outside the free layer due to *J*^STT^ and *J*^SOT^, simultaneously. Figures show the Oersted field due to only WP_STT_ (**d**), only WP_SOT_ (**e**) and hybrid (**f**) cases with the ratio *J*^SOT^/*J*^STT^ = 1000. Arrows show the direction while the arrow size along with the colour map defines the strength of the Oersted field (H_Oe_).

Here, ${\overrightarrow{{m}}}_{{free}}$ and ${\overrightarrow{{m}}}_{{fixed}}$ are the unit vector along the magnetization of free and fixed layers, respectively. ${\overrightarrow{{H}}}_{{eff}}$ is the effective field including the exchange, magneto-static, anisotropy and current-induced Oersted fields. *α* is the damping constant, *M*_S_ is the saturation magnetization and *t*_*F*_ defines the thickness of the free layer. ${\overrightarrow{{\tau }}}_{{STT}}$ is the exerted torque on the magnetization of the free layer generated by the current flowing from the fixed to the free layer. ${\overrightarrow{{\tau }}}_{{STT}}$ consists of two terms, the first one is Slonczewski-like torque and the second field-like torque, as described in Eq. 2. On the other hand, ${\overrightarrow{\tau }}_{{SOT}}$ represents SOT, which in the present work is acting on the magnetization of the free layer. Here, $\overrightarrow{\sigma }$ is the unit vector along the direction of spin polarization of current generated by SHE. STT and SOT current density (*J*^STT^ and *J*^SOT^) are associated with WP_STT_ and WP_SOT_ along *z* and *x* directions, respectively, as indicated in Fig. 1(a). The cell size is fixed to 1 × 1 × 1 nm^3^ for the free layer. In this simulation, the current dependent ${{b}}_{{J}}$ and ${\tau }_{{F}}$ terms related to field-like torques due to STT and SOT, respectively, are not included as its behavior has not been fully understood^27^. Experimental studies also suggested that field-like torque has no deterministic effect on the magnetization switching of p-MTJs^16,30^. However, it is incorporated in the module so that one can use it in the future.

## Results and Discussion

Figure 1(a) presents the schematic of the simulated tri-layer p-MTJ cell with WP_STT_ with additional WP_SOT_. The thickness of the free layer was 1 nm. All parameters used in these simulations are mentioned in Table 1. Various magnitudes of *J*^STT^ and *J*^SOT^ are used in these simulations in order to find their effect on the magnetization response. We have kept the pulse duration of WP_SOT_ at 1 ns throughout the simulations because of the stochastic nature of SOT while the pulse duration for WP_STT_ is at 10 ns (Fig. 1(b)). An effective Oersted field acts on the magnetization of the free layer, which is associated with the flowing electric currents and it may affect the magnetization dynamics of the free layer^27^. As the simulated geometry suggests that there are two Oersted fields present inside and outside of the free layer associated with WP_STT_ and WP_SOT_, respectively, as shown in Fig. 1(c). All the simulations have been performed for 0 K assuming perfectly aligned magnetization of free and fixed layers along the z-axis. It is impossible to run those simulations in such a condition. So, the initial misalignment of magnetization is expected to become from the effective Oersted field due to the flowing currents. It does not play any significant role in the switching because its strength is very weak^27^. Snapshots in Fig. 1(d–f) show the Oersted field due to only WP_STT_, only WP_SOT_ and both (or hybrid pulse), respectively, with a ratio *J*^SOT^/*J*^STT^ = 1000. The ratio is simply chosen to be large for the better visualization of the effective Oersted field. The Oersted field generated by the current in the p-MTJ structure is numerically calculated by the separated procedure, and its calculation is out of the scope of this article and will be published elsewhere.

**Table 1:** Input parameters used in the SOT-implemented OOMMF simulations.

Parameters	Numerical values	Description
*t*_*Free*_	1 nm	Thickness of free layer
*t*_*Fixed*_	2 nm	Thickness of fixed layer
*A*	20.0 × 10^−12^ A/m	Exchange constant
*M*_s_	1.1 × 10^6^ A/m	Saturation magnetization
*K*_u_: Free	9.3 × 10^5^ J/m^3^	Magnetic anisotropy (Free layer)
*K*_u_: Fixed	1.2 × 10^6^ J/m^3^	Magnetic anisotropy (Fixed layer)
Mesh size	1 × 1 × 1 nm^3^	Unit cell
temp	0 K	Absolute temperature
Pulse width	10 ns	Width of write pulse
*α*	0.02	Damping constant
*η*	0.7	Spin-torque efficiency
*θ*_*SO*_	0.12 for Ta	Spin-orbit torque efficiency

**Figure 2:**
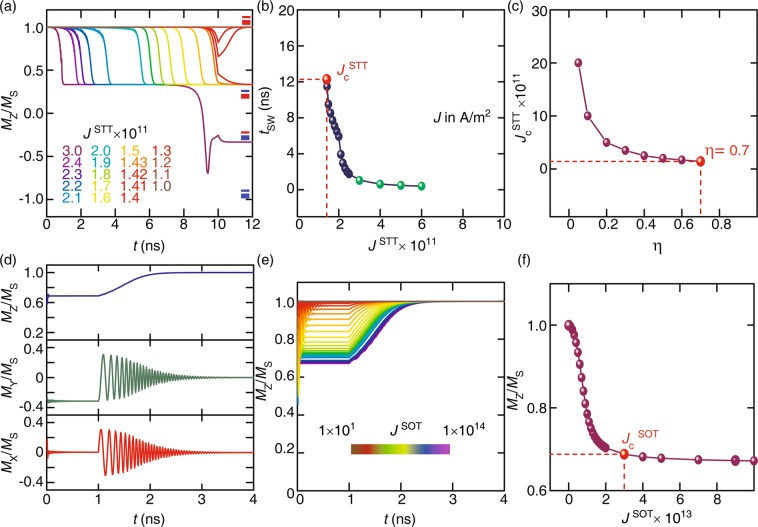
(**a**) The magnetization (*M*_Z_/*M*_S_) dynamics as a function of time under the influence of various WP_STT_ amplitudes, (**b**) corresponding *t*_SW_ as a function of *J*^STT^ and (**c**) effect of spin torque efficiency (*η*) on *J*_c_^STT^. The response of magnetization components under the influence of WP_SOT_ only is shown in (**d**), with the magnetization tilt as a function of *J*^SOT^ (**e**,**f**).

Figure 2(a) shows the magnetization behavior as a function of WP_STT_ with a duration of 10 ns at various *J*^STT^. The free layer is switched mainly through spin precessions on the order of 10^11^ A/m^2^ along the z axis, where the switching is judged to be accomplished when the z-component of magnetization (*M*_z_/*M*_s_) reaches to 0.33 from 1.00. The critical current density (*J*_c_^STT^) for which the magnetization of the free layer start to switch under only WP_STT_ is found to be 1.42 × 10^11^ A/m^2^ with the switching time (*t*_SW_) of 12.3 ns which is over the WP_STT_ duration (10 ns). The switching is proceeding under the *J*^STT^ till 10 ns through precessions and completed by ‘damped oscillations’ within the next 2.3 ns after WP_STT_ gets off (Fig. S1). The data presented in Fig. 2(b) illustrates the effect of *J*^STT^ on *t*_SW_ in the case of WP_STT_ only. *t*_SW_ decreases as *J*^STT^ increases as a result of strong torque. Figure 2(c) shows that change in *J*_c_^STT^ as a function of spin-torque efficiency (*η*): *J*_c_^STT^ increases as *η* decreases. Such a tendency was expected as spin efficiency decides the magnitude of generated torque which eventually varies *J*_c_^STT^. In these simulations, we set *η* = 0.7 and it can be varied for another system. Figure 2(d) shows the magnetization dynamics under the influence of only WP_SOT_. WP_SOT_ of 1 ns was implemented in the x direction in order to evaluate the minimum critical SOT current density (*J*_c_^SOT^). *J*_c_^SOT^ is the value of current density for which the magnetization becomes in-plane and *M*_z_/*M*_s_ reaches to 0.69 from 1.00 in the case of only WP_SOT_. The spin direction is defined in such a way that y spin should be accumulated at the interface between free and nonmagnetic polarizing layers. From the curve presented in Fig. 2(d) shows the effect of torque generated by the spin current on magnetization. Lower *J*^SOT^ shows a low tilt of magnetization and reflects that the small magnitude of *J*^SOT^ also regulates the magnetization. As a nature of SOT, it makes the magnetization in-plane within a very short time (depends on the magnitude of *J*^SOT^) as WP_SOT_ applied. But, it requires a very high current density nearly 3.0 × 10^13^ A/m^2^ which is expected as mentioned in literature^31^. Remember that we have used spin-orbit torque efficiency (*θ*_SO_) to be 0.12 found in Ta^17^. It has been deducted from these simulations that the sub-nano second WP_SOT_ is enough to align the magnetization along the in-plane direction. Once WP_SOT_ gets off, there is an equal probability of magnetization switching in either direction through damped oscillations to the original state or in the switched state which depends on the instantaneous state of magnetization at the end of the current pulse. Figure 2(e,f) demonstrate the tilting of magnetization as a function of *J*^SOT^. They suggest that even a very tiny value of *J*^SOT^ (10^2^ A/m^2^) can initiate the magnetization tilt and that can be utilize to save energy consumption.

**Figure 3:**
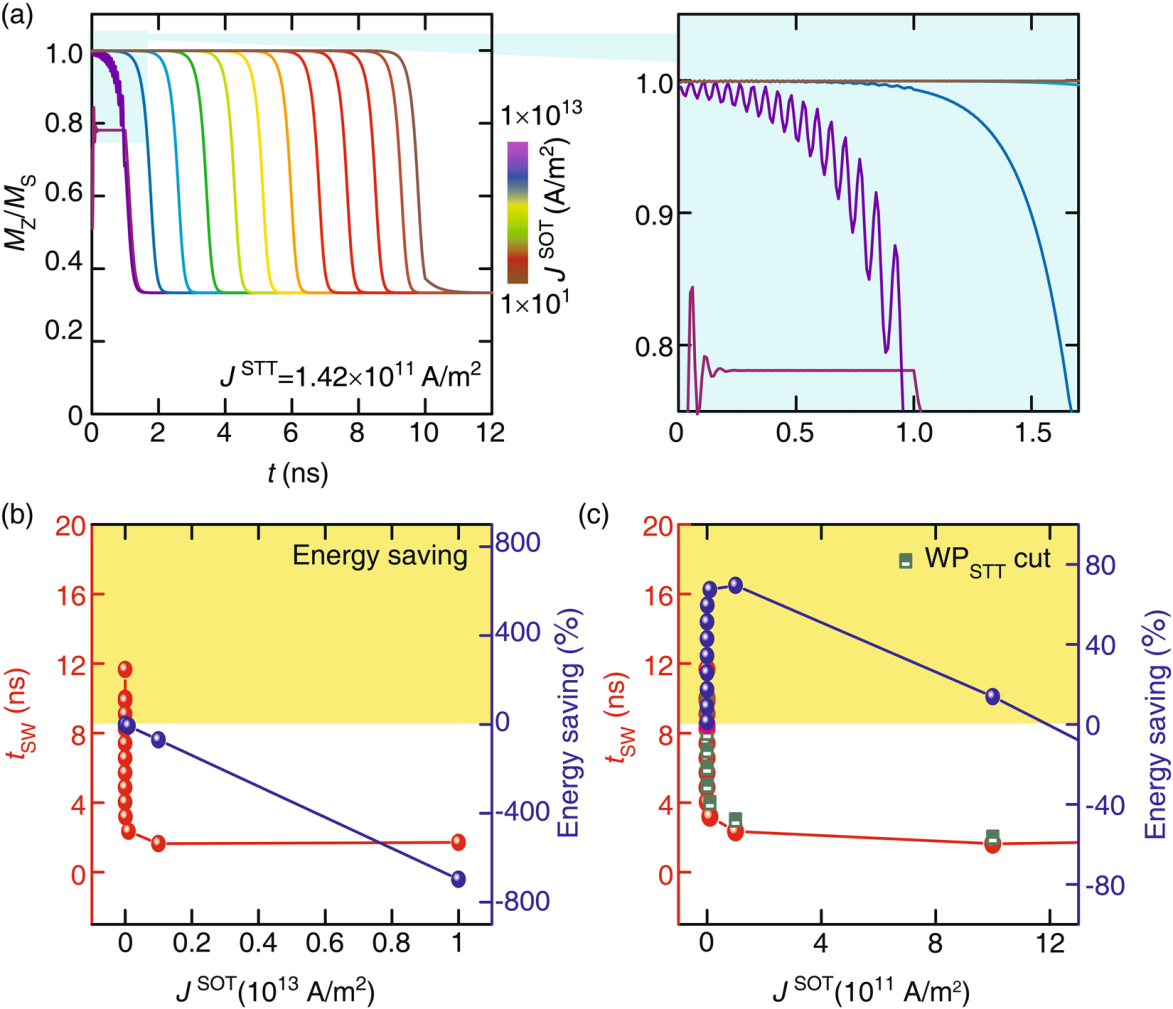
(**a**) The magnetization (*M*_Z_*/M*_S_) dynamics as a function of time under the influence of hybrid current with various WP_SOT_ amplitudes, (**b**) corresponding *t*_SW_ and energy consumption as a function of *J*^SOT^ and (**c**) energy saving on WP_STT_-cut correspond to its *t*_SW_ at *J*^STT^ = 1.42 × 10^11^ A/m^2^.

Figure 3(a) depicts that the effect of WP_SOT_ along with WP_STT_ on the magnetization dynamics of the free layer. It is clearly observed from the high *J*^SOT^ region in Fig. 3(a) that the switching is dominated by *J*^SOT^ through a direct mechanism in the duration of WP_SOT_ (i.e., 1 ns) while the switching of magnetization of the free layer is completed by *J*^STT^ through precession after WP_SOT_ gets off. The inset of Fig. 3(a) shows large precession which is occurred when *J*^STT^ and *J*^SOT^ are comparable to each other, e. g. WP_STT_ (1.42 × 10^11^ A/m^2^) and WP_SOT_ (1.00 × 10^11^ A/m^2^) acted simultaneously. Once WP_SOT_ gets off, the precession becomes small similar to the case of only WP_STT_. The contribution of each pulse (WP_STT_ and WP_SOT_) in the hybrid case is easily distinguishable in order to observe their effects because of the difference in the nature of two phenomena. This can be very helpful to tune the parameters of WP in order to save energy. Figure 3(b) shows *t*_SW_ and energy saving due to a decrease in the value of *J*^SOT^ at *J*^STT^ = 1.42 × 10^11^ A/m^2^. It is clearly observed that high values of *J*^SOT^ support to the reduction of *t*_SW_ but on the cost of write energy as there is additional WP_SOT_. A higher magnitude of *J*^SOT^ makes high energy consumption which is shown as the negative energy saving region in Fig. 3(b) and energy consumption increases with the value of *J*^SOT^. In such a case, *t*_SW_ is found to be reduced significantly but there is no energy saving. Energy can be saved by cutting WP_STT_ off immediately after the switching, as shown in Fig. 3(c). In the WP_STT_ cut-off case, the energy becomes saved until *J*^SOT^ increases to 12 × 10^11^ A/m^2^. In other words, it starts to save energy when *J*^SOT^ well bellow of 10^12^ A/m^2^. The energy saving becomes a maximum of 70% when *J*^SOT^ reaches 1.0 × 10^11^ A/m^2^. The simulated result in Fig. 3(c) also suggests that the energy is saved even if the applied value of *J*^SOT^ is nearly equal to ‘10^2^ A/m^2^’. It is worth to mention here that even such a tiny magnitude of *J*^SOT^ determines the initialization of switching in the case of hybrid switching which eventually helps to speed up the switching and save the energy. This study can be categorized into three important sections based on switching speed and energy consumption for the sake of convenience;

- **Fast switching with high energy consumption:** In Fig. 3(a), the value of *J*^SOT^ is high (on the order of 10^12^ A/m^2^) enough to make the magnetization in-plane in a fraction of second. We have kept this value lower than *J*_*c*_^SOT^ in order to make a better demonstration of an effect as higher current density is abandoned for the device purpose. In addition to the quick in-plane orientation of magnetization, *J*^SOT^ does not allow the magnetization of the free layer to acquire the switched state. Once WP_SOT_ stopped, the magnetization of the free layer tends to its switched state under the influence of *J*^STT^ and *t*_SW_ is decided by its value. Although, *t*_SW_ is too short but the magnitude of *J*^SOT^ does not support this region for energy saving as shown in Fig. 3(b).
- **Fast and energy efficient switching:** In this region, the order of *J*^SOT^ is kept between 10^2^ to 10^11^ A/m^2^ which provide the initial magnetization tilt in the case of only WP_SOT_ as shown in the Fig. 2(e–f). In this region, the magnetization tilt due to *J*^SOT^ defines the initial state on the application of WP_SOT_. Then, magnetization precesses under the influence of both WP_SOT_ and WP_STT_ for the duration of WP_SOT_, i.e., 1 ns. In this duration, the vector sum of the two torques ${\overrightarrow{\tau }}_{{\rm{STT}}}$ and ${\overrightarrow{\tau }}_{{\rm{SOT}}}$ acts on the magnetization of the free layer and re-defines the initial state for WP_STT_ after WP_SOT_. It causes less effort for *J*^STT^ to make magnetization switched and assists for energy saving on WP_STT_-cut. This region is dominated by the precessional switching and supports the largest energy saving because ${\overrightarrow{\tau }}_{{\rm{SOT}}}$ acts as complement of ${\overrightarrow{\tau }}_{{\rm{STT}}}$.
- **WP**_**SOT**_ **assistance only:** This region starts from the value of *J*^SOT^ below 10^2^ A/m^2^ at a fix *J*^STT^ of 1.42 × 10^11^ A/m^2^. In this region, the switching is accomplished mainly under the influence of *J*^STT^ as the magnitude of *J*^SOT^ is very small. Similar to the case at *J*_c_^STT^, the switching is completed by damped oscillations after WP_STT_. *J*^SOT^ below 10^2^ A/m^2^ (e.g., 10 A/m^2^) assists the STT switching but due to WP_STT_ cut-off at 10 ns, it is completed by damped oscillation in 11.66 ns (shown in Fig. S1). In this case, *t*_SW_ is found to be less as compare to only WP_STT_, i.e., 12.3 ns. Although, there is a certain effect of *J*^SOT^ on magnetization switching but it is unable to save energy due to the involvement of damped oscillations to complete switching and left no margin for WP_STT_-cut.

**Figure 4:**
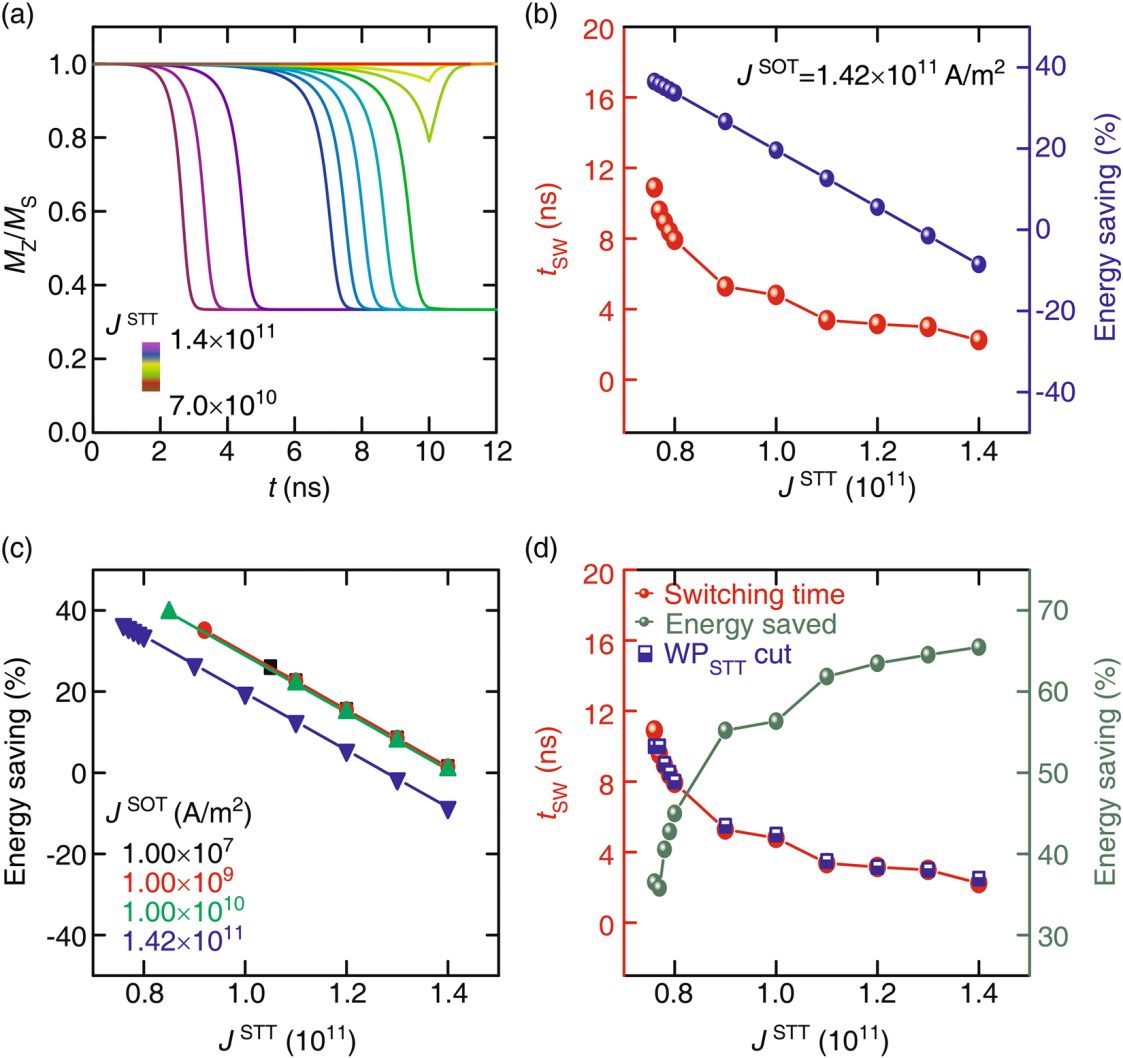
(**a**) The magnetization (*M*_Z_*/M*_S_) dynamics as a function of time under the influence of WP_STT_ with various *J*^STT^ at a fixed *J*^SOT^ of 1.42 × 10^11^ A/m^2^ and (**b**) corresponding dependency of *t*_SW_ and energy saving on *J*^STT^. (**c**) Energy saving as a function of *J*^STT^ for various *J*^SOT^. (**d**) *t*_SW_ and energy saving as a function of *J*^STT^ at *J*^SOT^ = 1.42 × 10^11^ A/m^2^ with WP_STT_-cut.

Figure 4(a) shows the effect of WP_STT_ on the magnetization dynamics of the free layer at a fixed current density *J*^SOT^ of 1.42 × 10^11^ A/m^2^. It is clear that *J*^STT^ can be reduced down to 0.7 × 10^11^ A/m^2^ with the complete magnetic switching accomplishment of the free layer. Figure 4(b) shows *t*_SW_ and energy saving as a function of *J*^STT^ for the fixed *J*^SOT^. One can save the energy up to 38% on reducing *J*^STT^ from 1.4 to 0.7 ( × 10^11^ A/m^2^) at *J*^SOT^ = 1.42 × 10^11^ A/m^2^. However, *t*_SW_ needs to be sacrificed to achieve such a high energy saving. The energy saving as a function of *J*^STT^ is demonstrated in the Fig. 4(c) for various values of *J*^SOT^ as it confirmed that a tiny amplitude of *J*^SOT^ affects the switching mechanism (shown in Fig. 3(a)). Energy consumption can be further reduced by cutting WP_STT_ off immediately after the corresponding *t*_SW_. Our results suggest that energy up to 66% can be saved if we cut WP_STT_ off right after *t*_SW_, as shown in Fig. 4(d). It is an important outcome of this research and can be considered as a highly rated perspective from the industrial point of view. As far as the switching process is concerned, there is not much difference in the magnetization dynamics except for the amplitude of precession. It is observed that the precession amplitude is found to be large in the particular combination of *J*^STT^ and *J*^SOT^ in the case of hybrid switching as compared to that in the case of only WP_STT_.

## Conclusions

In conclusion, we have investigated the magnetization behavior of the free layer under both WP_STT_ and WP_SOT_ for the p-MTJ cell in a dimension 20 nm using a newly coded STT-SOT hybrid torque module for the OOMMF micro-magnetic simulation package. The hybrid switching scheme employing both SOT and STT phenomena suggests that a very small magnitude of *J*^SOT^ affects the switching mechanism and assists to switch the magnetization quickly. As demonstrated in our simulations, WP_SOT_ influences STT switching in respect of writing-energy saving up to 70% along with the improved switching speed. Researchers have been optimistically looking for the engineering routes to reduce *J*_c_ because SOT itself takes high current density to switch the magnetization. Considering this fact, we demonstrated the application of WP_SOT_ where even a tiny amplitude of *J*^SOT^ facilitates the STT switching in order to save energy with fast switching in practical devices. Furthermore, our simulation results also provide an efficient way to resolve the high current issue in addition to write latency in STT-MRAM by WP_SOT_ implementation.

## Methods

For the micro-magnetic simulations, object oriented micro-magnetic framework (OOMMF) based on the Landau-Lifshitz-Gilbert-Slonczewski equation is used which also includes the spin orbit torque (${\overrightarrow{\tau }}_{{\rm{SOT}}}$) as a new torque term along with the spin-transfer torque (${\overrightarrow{\tau }}_{{\rm{STT}}}$). This equation was numerically solved using the fourth-order Runge-Kutta method. The simulated p-MTJ is composed of a Ta/CoFeB/MgO/CoFeB multilayer. The parameters considered in these simulations are given in Table 1.

## Supplementary information

Supportive Information.
