# Supplementary material for: Impact of Spin-Orbit Torque on Spin-Transfer Torque Switching in Magnetic Tunnel Junctions
Source: Sci Rep. 2020 Feb 18;10:2799. doi: 10.1038/s41598-020-59533-y (PMC7029010; doi:10.1038/s41598-020-59533-y)
Supplement: Supplementary file 1 — Supportive Information. [file 41598_2020_59533_MOESM1_ESM.docx]

**Supplementary Information**

**Impact of Spin-Orbit Torque on Spin-Transfer Torque Switching in Magnetic Tunnel Junctions**

*Sachin Pathak^1,2^, Chanyoung Youm^1^ and Jongill Hong^1,*^*

^1^Materials Science and Engineering, Yonsei University, Seoul 03722, Korea.

^2^Present Address: Physics, University of Petroleum and Energy Studies, Dehradun 248007, India.

**Contents:**

**S1: Comparison of magnetization switching of STT and STT-SOT hybrid**

*Corresponding authors: hong.jongill@yonsei.ac.kr

**S1. Comparison of the magnetization switching by STT and STT-SOT hybrid**

Figure S1 compares the magnetization dynamics (*M*_Z_/*M*_S_, *M*_Y_/*M*_S_ and *M*_X_/*M*_S_) between STT-SOT hybrid switching and only WP_STT_ where the values of *J*^SOT^ and *J*^STT^ are defined as 10 A/m^2^ and 1.42×10^11^ A/m^2^, respectively. In both cases, the switching is initiated with precession while accomplished by damped oscillations after the pulse-off at 10 ns. Although, they follow the similar magnetization dynamics to complete the switching but the significant effect of WP_SOT_ can be easily observed in the case of STT-SOT hybrid switching as compared that in the case of only WP_STT_. A speedy switching is initialized in the case of hybrid switching as compared to only WP_STT_ by magnetization precession within 10 ns. This can be attributed to the initial tilt due to WP_SOT_. Consequently, damped oscillations start at 10 ns and complete the switching at 11.66 ns for hybrid switching, while *t*_SW_ is 12.3 ns in the case of only WP_STT_. Although, the switching is observed faster as compared to that in the case of only WP_STT_ because of the additional WP_SOT_. However, this additional *J*^SOT^=10 A/m^2^ contributes to an increase in energy consumption.

**
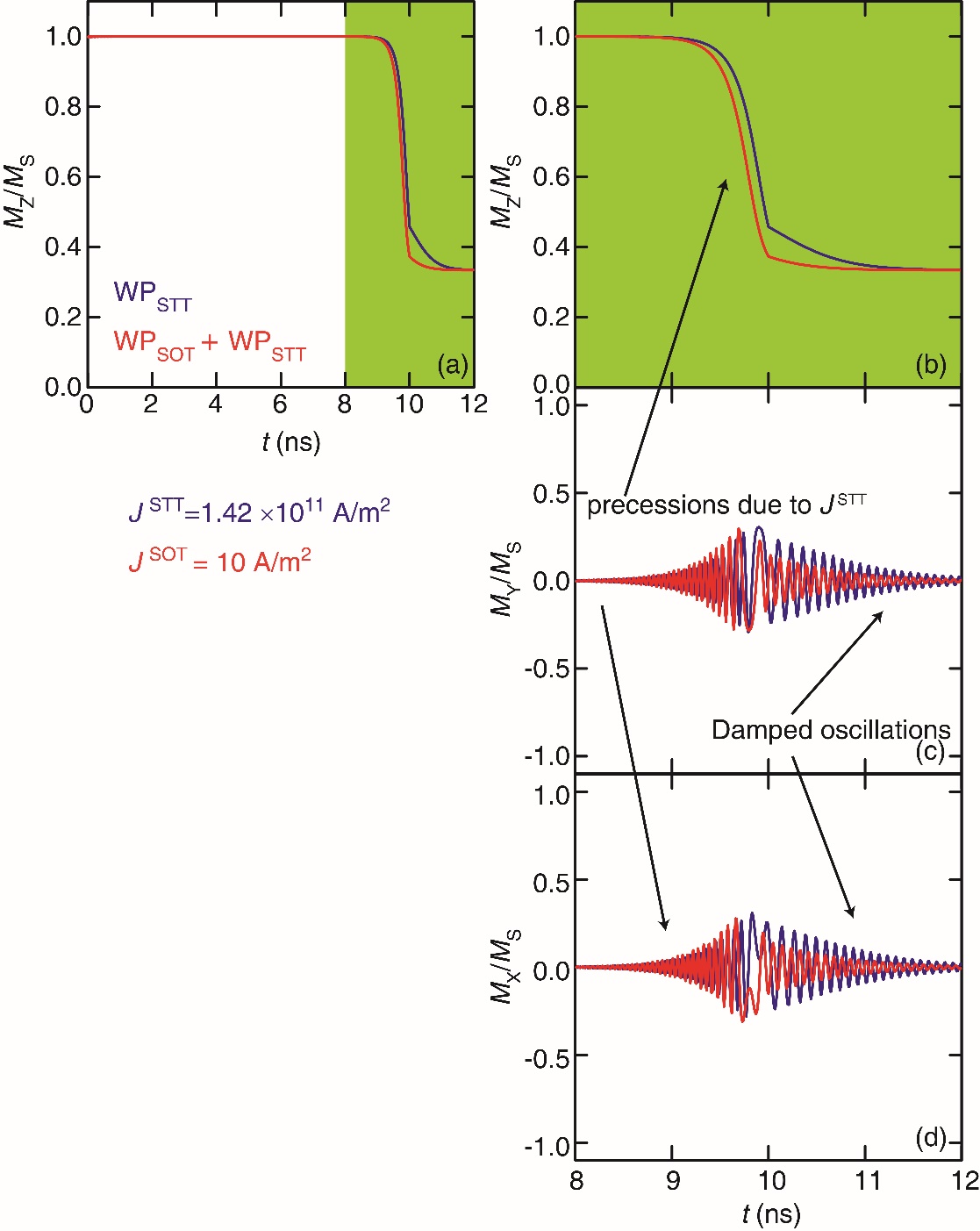
**

**Figure. S1.** Comparison of dynamics of the magnetization switching for WP_STT_ with *J*_c_^STT^=1.42×10^11^ A/m^2^ and for the hybrid STT-SOT pulse with *J*_c_^STT^=1.42×10^11^ A/m^2^ and *J*^SOT^=10 (or 1×10^1^) A/m^2^.
